# Analysis of Codman microcerebrospinal fluid shunt

**DOI:** 10.1002/brb3.1002

**Published:** 2018-09-11

**Authors:** Axel Sandvig, Kai Arnell, Jan Malm, Anders Eklund, Lars‐Owe D. Koskinen

**Affiliations:** ^1^ Department of Pharmacology and Clinical Neurosciences Division of Neuro, Head and Neck Umeå University Umeå Sweden; ^2^ Department of Neuromedicine and Movement Science Norwegian University of Science and Technology Trondheim Norway; ^3^ Department of Surgery Umeå University Umeå Sweden; ^4^ Department of Radiation Sciences Umeå University Umeå Sweden

**Keywords:** cerebrospinal fluid, clinical retrospective study

## Abstract

**Introduction:**

Ventriculo‐peritoneal cerebrospinal fluid (CSF) shunt is the most common method of treating pediatric hydrocephalus. The Codman microadjustable valve (CMAV) is a CSF shunt constructed for children. The objective of the study was (a) to analyze complications after insertion of a CMAV shunt in hydrocephalic children, (b) to analyze complications after replacing a CMAV by an adult‐type Codman Hakim adjustable valve shunt (CHAV), and to (c) analyze the in vitro characteristics of the CMAV shunt and correlate the findings with the clinical performance of the shunt.

**Methods:**

A retrospective study analyzed a cohort of hydrocephalic children who had received a CMAV shunt and later replaced by a CHAV shunt. We report on the complications that resulted from replacing the CMAV with the CHAV. We tested six CMAV shunts with or without an antisiphon device (ASD) in which opening pressure, resistance, sensitivity to abdominal pressure, ASD position dependency, and function were determined. The test results were correlated with the clinical performance of the shunt in the retrospective study.

**Results:**

Thirty‐seven children (19 boys, 18 girls) were identified. Within the first month after shunt placement, a total of 10 patients (27%) developed complications including infections, hygromas, and shunt dysfunction. Shunt survival varied from 1 week to 145 months. Over the 10‐year follow‐up period, 13 children had their shunts replaced, six of them with a CHAV without any further complications. A bench test of the CMAV was done to test whether the opening pressure was in agreement with the manufacturer's specifications. Our results were generally in agreement with specifications stated by the manufacturer.

**Conclusion:**

Replacing a CMAV with a CHAV was well tolerated by the patients. Bench test results were generally in agreement with manufacturers specifications. Replacing a CMAV with a CHAV in pediatric hydrocephalus patients can be accomplished safely.

## 1 INTRODUCTION

Ventriculo‐peritoneal cerebrospinal fluid (CSF) shunt implantation is the standard therapy for treating pediatric hydrocephalus. Shunt malfunction is a major problem in the pediatric population. A shunt malfunction can be subgrouped into shunt over‐ or underdrainage (Faulhauer & Schmitz, 1978; McCullough, 1986), shunt obstruction (Becker & Nulsen, 1968; Arnell & Olsen, 2004), valve failure (Lundar, Langmoen, & Hovind, 1991), shunt infection (Nulsen & Becker, 1966), or due to growth of the children (Tsingoglou & Forrest, 1968). Nonadjustable shunts may have an increased risk of over‐ or underdrainage (Arnell, Eriksson, & Olsen, 2006) with risk of reoperation. Furthermore, it has been documented that using a nonadjustable shunt increases the risk of proximal shunt obstruction requiring revision (McGirt et al., 2007). However, a multicentre prospective randomized control study that included 377 patients found no statistical difference between an adjustable valve (CHAV) and conventional valve shunts in terms of shunt failure that required revision (Pollack, Albright, & Adelson, 1999).

The modern CSF shunt is a complex construction with or without separate antisiphon device (ASD). Important features are the possibility of changing the opening pressure noninvasively to obtain the optimal opening pressure and if needed, treat over‐ or underdrainage (Arnell et al., 2006; Rohde, Mayfrank, Ramakers, & Gilsbach, 1998; Zemack, Bellner, Siesjö Strömblad, & Romner, 2003). Several CSF shunts with adjustable opening pressure are commercially available and an increasing number of studies have evaluated the clinical benefits and basic hydrodynamics of these shunts (Boon et al., 1998; Czosnyka, Czosnyka, & Pickard, 1998; Deininger & Weyerbrock, 2009; Drake et al., 1998; Ekstedt, 1978; Kestle et al., 2000; Lundkvist, Eklund, Koskinen, & Malm, 2001, 2003; Malm et al., 1995). Some CSF shunts have been tested in independent laboratories in order to validate the manufacturer's specifications (Arnell, Koskinen, Malm, & Eklund, 2009; Czosnyka et al., 1998; Lundkvist et al., 2003). An example of the importance of validation of different shunt systems by independent laboratories was demonstrated in a study where retrograde flow was found in an approved CSF shunt (Eklund, Koskinen, & Malm, 2004). Despite these efforts, little progress has been made over the last decades in preventing or reducing shunt failures (Sherman & Wensheng, 2008).

The Codman Micro™ adjustable shunt (CMAV; Codman, Johnson & Johnson Co., Raynham, MA, USA) has a small size and is especially designed for use in children. According to the manufacturer, it has the same specifications as the Codman Hakim adjustable valve (CHAV). However, the smaller size could make it more difficult to locate the valve without radiological verification when the valve needs adjustment.

The CMAV has been the first choice of shunt for children in our department since 1997 because of its smaller size compared with the CHAV. However, in those children that had their CMAV shunt revised with a CHAV, we often noted a problem of achieving an optimal opening pressure. This motivated us to do a retrospective quality assessment study of those children given a CMAV as their first shunt between 1997 and 2007, and in which we also analyzed risk, complications and clinical outcome in the cohort of patients that revised their CMAV shunt with a CHAV. We also wanted to test whether the problems we experienced could be due to the valve function and endurance of the CMAV. This question was addressed in an in vitro bench test of the CMAV in which we compared our results with those obtained in an earlier test of the CHAV (Arnell et al., 2009) and those given by the manufacturer.

## 2 MATERIAL AND METHODS

### 2.1 Clinical

This study was approved by the regional ethical committee (Dnr 2016/162‐31). The medical records of all children, shunted with a CMAV as first shunt at the Department of Neurosurgery, Umeå University Hospital, between May 1997 and December 2007, were reviewed in terms of etiology, age at shunting, location of the ventricular catheter, adjustments, shunt survival, and reason for shunt revision. Shunt revisions in which the CMAV was replaced with a CHAV shunt, as well as the number of adjustments and any relevant clinical symptoms that may have resulted from the procedure were analyzed. Minor complications were defined as those that only needed an adjustment or antibiotic treatment. A serious complication was defined as a shunt that needed either a revision or a replacement. Postoperative complications were defined as those occurring within 1 month of surgery.

### 2.2 Experimental evaluation

Six new CMAV shunts (kind gift from Codman, Johnson & Johnson Co) with and without the corresponding ASD (Siphonguard™) were tested in vitro with the original proximal (14 cm) and distal catheters. We used an automated computerized experimental setup to evaluate the Codman Micro™ shunt with and without a corresponding ASD. Before the tests, all shunts were soaked in deaerated water for at least 24 hrs and simultaneously perfused with 0.33 ml/min deaerated water. The shunts and ASD were kept in a water bath between the different tests.

The setup for shunt testing has previously been described (Eklund et al., 1999, 2004; Lundkvist et al., 2003; McCullough, 1986). Briefly, a computer (Apple Computer Inc. Cupertino, CA, USA) controlled the fully automated device including regulation of pressure and collection of data. The CSF shunt was mounted on a horizontal plate and submerged into 10 cm of water to simulate the subcutaneous tissue pressure. Reference pressures such as zero pressure level, and levels simulating siphoning pressure (−30 cm H_2_O, −22.1 mmHg) and the abdominal pressure (8.7 cm H_2_O, 6.4 mmHg), were obtained by leading the outflow from the shunt system to an overflow container with a constant water level.

The in‐house developed software (using Lab view; National Instruments, Austin, TX, USA) regulated the simulated ICP according to a triangular waveform with duration of 60 min. The pressure was regulated between zero and the lowest multiple of 500 Pa (3.8 mmHg) resulting in a maximum flow exceeding 1.2 ml/min. The procedure was repeated a minimum of four times for each measurement. Measurements obtained during cycles disturbed by air bubbles were excluded.

Every shunt was tested at its lowest, middle, and highest opening pressure (30, 100, and 200 mm H_2_O) for analysis of opening pressure and resistance with and without an abdominal hydrostatic component. One CMAV shunt with a performance setting of 30, 100, and 200 mm was tested with six different Siphonguards™ placed at +10 cm above and −30 cm below the ventricular catheter tip. The opening pressure and resistance were determined at all positions and with siphoning test at −30 cm.

### 2.3 Statistics

Statistics was performed using JMP 10.0 (SAS Institute Inc., Cary, NC, USA). Values are expressed as mean ± standard deviation (*SD*). Paired *t* test was used to test for differences between groups. *p* ≤ 0.05 was considered statistically significant, and Bonferroni correction adjusting for multiple tests was used.

## 3 RESULTS

### 3.1 Clinical

**Figure 1:**
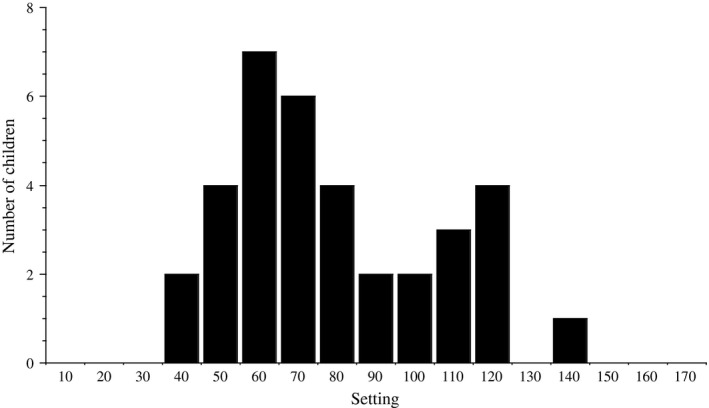
Setting in mm H2O at shunt insertion. For two of the 37 patients included in the study, no data have been documented in the patient journals concerning their shunt settings. Thus, the figure only includes data from 35 patients

During the study period, 37 hydrocephalic children (19 boys and 18 girls) had a CMAV inserted. In our material, we found that all the patients who received a CMAV were less than 1 year of age at the time of surgery, even though age <1 year was not exclusion criteria. The etiology for hydrocephalus was myelomeningocele (*n* = 11), preterm birth with postnatal hemorrhage (*n* = 11), aqueductal stenosis (*n* = 8), and other (*n* = 7). The shunt was inserted at the age of 1 day to 11 months (median 2 months). The shunt was inserted through a frontal burr hole in 23 children. An occipital or parietal approach was used in 10 and 3 children, respectively. For one child, data were missing regarding location of the burr hole. The setting at shunt insertion varied between 40 and 140 mm H_2_O (mean 78 mm) for 35 children. The most common settings were 60 mm H_2_0 (7 patients) followed by 70 mm H_2_0 (6 patients). For two patients, data were missing (Figure 1).

**Figure 2:**
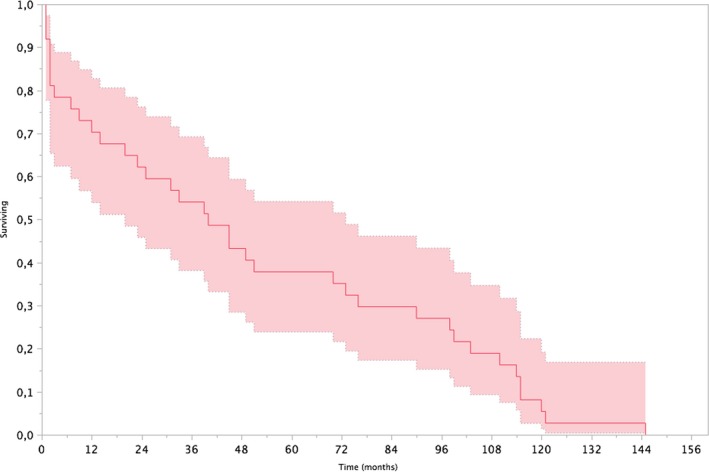
Kaplan–Meier cumulative survival plot of the shunt. The red line represents the median shunt survival in months. The shadowed area represents the range for shunt survival at each time point

The follow‐up was at 12–145 months (median 45 months). During this period, six of the patients had died (mors subita, tumor cerebri, multiple malformations, pneumococcus–pneumonia with shunt infection, prematurity, and hemorrhage). The shunt survival varied between 1 and 145 months (median 39 months; Figure 2).

### 3.2 Complications

Within the first postoperative month, 10 complications (27%) were documented (Table 1). Hygroma and subcutaneous CSF leakage were treated with valve adjustments of the shunt. During the first 6 months, the opening pressure was adjusted in 28 of 37 patients (76%) between one and six times.

**Table 1:** Complications within 1 month after shunt insertion and treatment

Complications	Number	Treatment
Hygroma	3	Adjustment
Shunt infection	2	Reoperation
Extra‐abdominal catheter	1	Reoperation
Y‐connector catheter other side	1	Reoperation
Necrosis	1	Reoperation
Pressure wound/infection	1	Antibiotics
Vomiting	1	Medication
Total	10	

During the follow‐up time, a total of 59 valve adjustments were performed and in 11 of these adjustments (19%) there were difficulties. In nine of these cases, the placement of the valve had to be identified with X‐ray before adjustment, and in two other patients, there were several unsuccessful attempts before the valve was correctly adjusted.

### 3.3 Revisions and replacements

During the follow‐up period, of 145 months, a total of 22 patients (59%) of 37 underwent either a shunt revision in which a defective part of the shunt was exchanged (*n* = 9; 24%) or received a new shunt (*n* = 13; 35%) with a median 5 months to replacement (range between 1 and 113 months). The revision was due to proximal obstruction (*n* = 6), distal obstruction (*n* = 2), and a dysfunctional y‐connection (*n* = 1). Shunt replacement was due to infection (*n* = 4; 31%), dysfunction of the valve (*n* = 3; 23%), proximal obstruction (*n* = 2; 15.5%), and distal obstruction (*n* = 2; 15.5%). In five of these children, the shunt was exchanged to a CHAV, in four cases to a new CMAV. One of those CMAV shunts was later replaced with a CHAV. The other four patients had Strata (*n* = 1), standard Hakim (*n* = 1), or ventricular abdominal catheter inserted (*n* = 2).

Of the six children changing from CMAV to CHAV, two experienced a persistent headache (had headache prior to change) that did not respond to any subsequent changes in the opening pressure. Of the other patients, one developed a hygroma postoperatively, which was successfully treated with adjustment of the valve (Table 2).

**Table 2:** Symptoms and adjustments after changing from CMAV to CHAV in six patients

Patients	Symptoms	Adjustments
1	Headache, hyperactivity	2
1	Headache	1
1	Hygroma	2
1	No	2 (pre‐op overdrainage treated)
1	No	0 (regress of pre‐op hygroma)
1	No	0

CHAV, Codman Hakim adjustable valve shunt; CMAV, Codman microadjustable valve.

### 3.4 Results of the laboratory in vitro test

The opening pressure flow curves were generally continuous and smooth, but the closing was discontinuous in steps. The opening pressure correlated with the performance‐level settings. According to the manufacturer's specification, the opening pressure in the CMAV at the lowest, medium, and highest setting was 2.2, 7.4, and 14.8 mmHg, respectively. The opening pressure in vitro at the medium and highest settings was in accordance with the manufacturer's specification; however, at the lowest setting it was twice that provided by the manufacturer (Table 3).

**Table 3:** Static opening pressure and resistance with and without abdominal pressure for CMAV at different performance levels. All data for simulated lying position

Setting in mm H_2_O (mmHg)	−Abdominal pressure		+Abdominal pressure	
	Opening pressure, mmHg	Resistance, mmHg/ml/min	Opening pressure, mmHg	Resistance, mmHg/ml/min
30 (2.2)	4.4 ± 1.9	3.9 ± 0.0	11.2 ± 2.9	4.1 ± 0.0
100 (7.36)	8.4 ± 0.6	3.7 ± 0.1	14.1 ± 1.0	3.8 ± 0.2
200 (14.7)	14.5 ± 2.6	3.7 ± 0.7	18.9 ± 6.0	3.7 ± 0.3

The simulated abdominal pressure resulted in a significant increase (*p* < 0.05) in the opening pressure (Table 3). The CMAV resistance was not dependent on the performance level or the abdominal pressure and was shown to be just beneath 5 mmHg/ml/min, which was regarded as the lower limit of the physiological resistance interval (Ekstedt, 1978). There were individual variations regarding opening pressure and resistance in the shunts. The variations in the opening pressure at the three settings ranged between 0.6 and 2.6 mmHg without abdominal pressure and 1.0–6.0 mmHg with simulated abdominal pressure. In resistance, the variations at different settings were small (ns) with <0.7 mmHg/ml/min, respectively, <0.3 mmHg/ml/min. In simulated laying position of the patient, the position of the SiphonGuard did not affect the opening pressure or the resistance (Table 4).

**Table 4:** Static opening pressure and resistance for CMAV with the antisiphon device at position +10 and −30 cm. All data are for simulated lying position

Setting in mm H_2_O (mmHg)	+10 cm		−30 cm	
	Opening pressure, mmHg	Resistance, mmHg/ml/min	Opening pressure, mmHg	Resistance, mmHg/ml/min
30 (2.2)	3.5 ± 0.2	5.6 ± 0.1	3.5 ± 0.1	5.6 ± 0.1
100 (7.36)	7.9 ± 0.6	5.5 ± 0.1	8.4 ± 1.1	5.6 ± 0.1
200 (14.7)	16.5 ± 0.8	5.4 ± 0.2	16.5 ± 0.8	5.4 ± 0.2

The siphoning, test in simulated sitting or standing position, showed that the resistance through the second outflow pathway was 10‐fold what was recorded for the primary outflow pathway (Table 5). This is in agreement with the manufacturer's specification.

**Table 5:** Siphoning test in CMAV and with the resistance the first and second way in mmHg/ml/min

Setting mm H_2_O (mmHg)	First way	Second way
30 (2.2)	4.5 ± 0.5	55.9 ± 3.7
100 (7.36)	4.9 ± 0.2	52.4 ± 5.6
200 (14.7)	4.6 ± 0.1	61.9 ± 5.9

## 4 DISCUSSION

The results presented in this study will be discussed in two parts. First, the results of the retrospective clinical cohort study will be evaluated followed by the laboratory conducted in vitro bench test study.

### 4.1 Adjustable CSF shunts

Both the CMAV and the CHAV are adjustable shunts. The CMAV is especially designed for children. However, our data indicate that it is quite difficult to find the exact location of the valve by palpation using the CMAV shunt as compared to the CHAV shunt. As a result, this often necessitates an X‐ray of the exact position of the CMAV valve before adjustment of the valve settings. In contrast, the CHAV valve is easily palpated on the patient skull something that permits the valve settings to be adjusted without first having to locate the position of the valve with X‐ray. The advantage of this is that children will be less exposed to X‐rays during a sensitive period of brain development. Another advantage of the CHAV compared with the CMAV is the shape and surface morphology of the valve house with less sharp edges in the CHAV. As discussed by Arnell et al. (2006), this may reduce the risk of pressure necrosis of the skin being stretched over the shunt valve house (Arnell et al., 2006). An alternative type of adjustable differential valve is the proGAV (Miethke‐Aesculap, Germany) that includes a gravitational unit. This valve has the major advantage of not requiring adjustments after MRI. Using this system, Gebert, Schulz, Haberl, and Thomale (2013) reported that in 47.7% of their patients, no adjustments after surgery were necessary while in 42% of the readjusted patients one adjustment sufficed to deal with radiological and clinical changes (Gebert et al. (2013)). In 30% of the readjusted patients, two adjustments were done while 19% of the readjusted patients received more than two adjustments (Gebert et al. (2013)). This compares with 76% of the patients in our own study that received one to six adjustments during the first 6 months postsurgery. This may partially reflect the fact that the CMAV and CHAV valves are MRI sensitive and therefore often need adjustments following an MRI control. Out of a total of 59 adjustments performed during the study period, 9 (15.3%) experienced problems identifying the valve necessitating the use of X‐ray for identification. Increased use of X‐ray for valve identification in a pediatric patient population is an added worry compared with a mechanical valve design such as the proGAV.

### 4.2 Complications

Six pediatric patients had their CMAV shunt revised and replaced by a CHAV shunt. Two of these patients continued to experience headaches in the postoperative period, which were not amendable despite several adjustments of the valve resistance. However, it is difficult to conclude that the headache was related to the type of shunt because they had headache before revision and of the small number of patients affected. In addition, the difference in resistance was insignificant when the CMAV was compared with the CHAV. Furthermore, we found no shunt dysfunction that could explain the headaches. The usefulness of an adjustable shunt in our pediatric patient population is illustrated by the fact that valve adjustments corrected both postoperative hydrocephalus and CSF leakage and thus improved the patients’ symptoms. Furthermore, even though repeated adjustments were performed, in some patients no further surgery was necessary supporting the argument for using valve‐adjustable shunts. This is particularly important in pediatric patients where repeated surgeries can negatively impact development and cognitive functions. Other studies have indicated the same benefit of using valve‐adjustable shunts (Gebert et al. 2013; Haberl et al., 2009; Rhode et al., 2009).

### 4.3 Shunt infections

Postoperative infections constitute a major complication in CSF shunting in the treatment of children with hydrocephalus. In our retrospective study, the infection rate in the first postoperative month necessitating surgical revision was 5.4% (2/37) with an overall 10‐year incidence of infections of 16.2% (6/37). Other studies have documented infection rates of 10.9% (14/128), 6.2% (54/884) acute, and 7.4% (65/884) overall that required revision (Borgbjerg, Gjerris, Albeck, & Børgesen, 1995; Kast, Duong, Nowzari, Chadduck, & Schiff, 1994). George, Leibrock, and Epstein (1979) showed that postoperative shunt infections are highest among the pediatric population (13.7% between 0 and 1 year) and in the elderly (17.1% between ages 61 and 70 years) with the highest infection rates (15.2%) in children occurring between 3 and 4 years of age (George et al., 1979). Arnell, Cesarini, Lagerqvist‐Widh, Wester, and Sjölin (2008) documented an overall incidence of infections after shunt placement in pediatric hydrocephalic patients of 8.2% covering a period of 13 years and which included a total of 474 shunt operations in 237 hydrocephalic children (Arnell et al., 2008). In their study, only three experienced pediatric surgeons performed the shunt operations, which may have contributed to the relatively low rate of shunt infections. In support of this, Borgbjerg et al. (1995) showed that infection rates correlate inversely with the experience of the neurosurgeon, with neurosurgical trainees having a significantly higher infection rate than their more experienced colleagues (Borgbjerg et al., 1995). Similar results were reported by Smith, Butler, and Barker (2004) and Choux, Genitori, Lang, and Lena (1992). Furthermore, a retrospective cohort study that included 431 children, of which 83% were <1 year of age, the average infection rate following shunt surgery was 22% (Ammarati & Raimondi, 1987). In this study, the youngest children had the highest infection rates, confirming the results of George et al. (1979). The discrepancy in infection rates between the various studies may be due to differences in the age and the size of the patient population as well as the length of the investigation period. Our own study includes a small number of patients. This may result in a biased reporting of higher infection rates. Although the rates of shunt infection vary between studies, it constitutes a major complication in the pediatric patient.

### 4.4 Mortality in the shunted cohort of pediatric patients

In our retrospective study, we found a total mortality rate of 16.2% over the follow‐up period of 10 years. This is similar to data from a larger retrospective study by Keucher and Mealey (1979) including 228 patients. In their study, the mortality rates in those patients with postoperative infection following shunt surgery were 18.0%, and in those patients without postoperative infection, the mortality rate was 15.7%. The overall total mortality rates were 37/228 (16.2%) including both patients receiving ventriculoatrial and ventriculo‐peritoneal shunts (Keucher & Mealey, 1979). In another study by Folitz and Shurtleff (1963), 113 hydrocephalic children with and without operation were compared over a 5‐year period. The mortality rate in the operated group was 15 of 65 (23%) and in the nonoperated group 22 of 48 (46%) died (Folitz & Shurtleff, 1963). Thus, in this study, it was documented clearly that hydrocephalic children that are not operated have twice the mortality rates as those receiving a shunt. The reduction in mortality rates documented in later studies may be due to a more rapid diagnosis, improvements in shunt technology, reduction in infection rates, and postoperative care.

### 4.5 Shunt survival

The shunt survival in our study ranged from 1 to 145 months (30–4,350 days; Figure 2). This is similar to other reported results (Kahn, Shamin, Rehman, & Bari, 2013; Wu, Green, Wrensch, Zhao, & Gupta, 2007). One study documented shunt survival in a pediatric patient population in the range of 11–1,058 days with a median time to first shunt failure of 68 days with an 18.5% shunt failure rate at 1 year (Kahn et al., 2013). However, no information was given about the type of shunts used. Other studies report similar findings with a 25% shunt failure rate (Wu et al., 2007). In comparison, we report a shunt revision rate of 24% and a replacement of 35%. Thus, the low shunt survival time is still a major problem in pediatric neurosurgery.

### 4.6 Bench test study

In the second part of our study, we analyzed the outflow resistance and compared the values of the CHAV and CMAV with the manufacturer's data. Furthermore, we tested the antisiphon properties of both the CHAV and the CMAV using a SiphonGuard and compared the results with those of the manufacturer.

### 4.7 Outflow resistance

In the clinical assessment of normal pressure hydrocephalus (NPH) patients outflow resistance is considered to be a positive predictor of improvement following shunt surgery. By lowering outflow resistance, shunt placement has a pronounced effect on the CSF dynamics (Malm, Lundkvist, Eklund, Koskinen, & Kristensen, 2004; Qvarlander, Lundkvist, Koskinen, Malm, & Eklund, 2013; Qvarlander, Malm, & Eklund, 2013; Qvarlander, Sundström, Malm, & Eklund, 2013). In this study, we addressed the issue of how changes in opening pressure and simulated abdominal pressure of an ASD affect the resistance of the CMAV when tested in vitro. In the bench test, we found that the static opening pressure of the CMAV shunt was similar to the specifications given by the manufacturer at the middle and highest settings, but higher than those given for the lowest opening pressure. In a study by Arnell et al. (2009), the opening pressure of the CHAV, subjected to a bench test, found similar pressures to what we report for the CMAV (Arnell et al., 2009). When the abdominal pressure was simulated in the in vitro test, we found significantly increased opening pressure. These findings are supported by similar test data reported for the in vitro testing of the CHAV shunt (Arnell et al., 2009). However, it is important to note that there are variations in the individual CSF shunts. Thus, the opening pressures on a specific setting varied between shunts. This is in agreement with other reports (Arnell et al., 2009). Thus, in the clinical setting, it is important to bear in mind that both the shunt's individual opening pressure and the intra‐abdominal pressure will affect the real opening pressure.

The in vitro testing of the CMAV also documented that the variations in resistance were insignificant at the different performance settings with and without abdominal pressure. This is in agreement with previous findings in which the CHAV was tested in vitro with increasing opening pressures at higher performance settings, while the variations in resistance were insignificant in the range of 0.1–0.4 mmHg/ml/min (Arnell et al., 2009). Thus, we conclude that from a CSF dynamic perspective the CMAV and CHAV had similar characteristics.

### 4.8 Antisiphon device

Slit ventricles may be a complication after shunt placement and occur as a result of a hydrostatic siphoning effect when the patient is in upright position. Using a shunt with an ASD may help avoid this problem. To test this, we used a SiphonGuard in which the flow is regulated with two pathways, and which according to the manufacturer, the flow should be reduced by a factor of ten when only the second pathway is open. The siphoning test results of the CMAV documented an approximate 10‐fold increase in the resistance in the second way pathway of the SiphonGuard compared to the first way, which is in agreement with the manufacturer's specifications. Furthermore, the results are comparable to similar tests performed on the CHAV (Arnell et al., 2009).

In testing the CMAV with SiphonGuard, we found higher opening pressures with increasing performance settings. The resistance, however, changed minimally between 5.4 and 5.6 mmHg/ml/min. This is in agreement with previous reports in which the CHAV was similarly tested. In that study, the opening pressure increased as the performance settings increased, while the resistance remained stable with a minimal variation between 5.7 and 5.9 mmHg/ml/min (Arnell et al., 2009). Our results show that neither the opening pressure nor the resistance changed for the CMAV with the SiphonGuard. This indicates that the ASD can be put in place already during the first operation.

## 5 CONCLUSION

The CMAV was easy to handle, but the valve settings were difficult to identify at adjustments. The shunt survival rates were similar to other reported series. The replacement of a CMAV shunt with a CHAV shunt was generally well tolerated, but our cohort was small and caution in the assessment is therefore necessary. The CMAV had at the lowest setting an opening pressure which was double that reported by the manufacturer. The resistance was not dependent on the performance level or the abdominal pressure. Since the ASD did not affect either the opening pressure or the resistance in lying position, it may be an advantage to implant a shunt with an integrated ASD at the first shunt insertion also in pediatric patients. Bench test studies validate the correctness of the manufacturer's reports on the performance of the various shunt systems. This is, however, an in vitro study that limits our ability to draw conclusion about the in vivo functions of the shunt systems. Keeping this limitation of the study in mind, we nevertheless believe it is relevant to compare such data with clinical outcome studies as we have done in this report.

## CONFLICT OF INTEREST

All authors certify that we have no affiliations with or involvement in any organization or entity with any financial or nonfinancial interests.
